# Metabolic flux analysis of 3D spheroids reveals significant differences in glucose metabolism from matched 2D cultures of colorectal cancer and pancreatic ductal adenocarcinoma cell lines

**DOI:** 10.1186/s40170-022-00285-w

**Published:** 2022-05-16

**Authors:** Tia R. Tidwell, Gro V. Røsland, Karl Johan Tronstad, Kjetil Søreide, Hanne R. Hagland

**Affiliations:** 1 Department of Chemistry, Bioscience and Environmental Engineering, University of Stavanger, Stavanger, Norway; 2 Department of Biomedicine, University of Bergen, Bergen, Norway; 3 Department of Oncology and Medical Physics, Haukeland University Hospital, Bergen, Norway; 4 Department of Gastrointestinal Surgery, Stavanger University Hospital, Stavanger, Norway

**Keywords:** 2D cell culture, 3D cell culture, Metabolism, Mitochondria

## Abstract

**Background:**

Most in vitro cancer cell experiments have been performed using 2D models. However, 3D spheroid cultures are increasingly favored for being more representative of in vivo tumor conditions. To overcome the translational challenges with 2D cell cultures, 3D systems better model more complex cell-to-cell contact and nutrient levels present in a tumor, improving our understanding of cancer complexity. Despite this need, there are few reports on how 3D cultures differ metabolically from 2D cultures.

**Methods:**

Well-described cell lines from colorectal cancer (HCT116 and SW948) and pancreatic ductal adenocarcinoma (Panc-1 and MIA-Pa-Ca-2) were used to investigate metabolism in 3D spheroid models. The metabolic variation under normal glucose conditions were investigated comparing 2D and 3D cultures by metabolic flux analysis and expression of key metabolic proteins.

**Results:**

We find significant differences in glucose metabolism of 3D cultures compared to 2D cultures, both related to glycolysis and oxidative phosphorylation. Spheroids have higher ATP-linked respiration in standard nutrient conditions and higher non-aerobic ATP production in the absence of supplemented glucose. In addition, ATP-linked respiration is significantly inversely correlated with OCR/ECAR (*p* = 0.0096). Mitochondrial transport protein, TOMM20, expression decreases in all spheroid models compared to 2D, and monocarboxylate transporter (MCT) expression increases in 3 of the 4 spheroid models.

**Conclusions:**

In this study of CRC and PDAC cell lines, we demonstrate that glucose metabolism in 3D spheroids differs significantly from 2D cultures, both in terms of glycolytic and oxidative phosphorylation metrics. The metabolic phenotype shift from 2D to 3D culture in one cell line is greater than the phenotypic differences between each cell line and tumor source. The results herein emphasize the need to use 3D cell models for investigating nutrient utilization and metabolic flux for a better understanding of tumor metabolism and potential metabolic therapeutic targets.

**Supplementary Information:**

The online version contains supplementary material available at 10.1186/s40170-022-00285-w.

## Background

Cellular metabolism is tightly controlled and serves an essential function for normal growth and survival. Since the identification of an altered metabolic state in cancer by Warburg almost 100 years ago [1], the importance of cancer metabolism for the understanding of tumor biology [2] and its potential for clinical targeting has been recognized [3]. The complexity of cancer metabolism has become a major research area [4], contributing to many studies investigating the metabolic heterogeneity of tumors [5] mainly driven by perfusion-limited nutrient access [6]. Common methods used to investigate cancer metabolism range from metabolomics and gene expression to protein expression, and live metabolic flux analysis. However, metabolic flexibility, with regard to nutrient stress, is best studied in real time. The cellular growth conditions and ability to adapt to nutrient stress has an effect on cells’ response to drug treatment [7].

Notably, the majority of cancer drug testing has been done in 2D cultures [8]. However, 3D cancer cell models are proposed as improved models for initial drug screening [9], based on their ability to model cell-cell interactions and natural nutrient gradients occurring in an avascular or poorly vascularized tumor microenvironment [9–11]. Therefore, it is important to study how growth and metabolism differ between the conventional 2D models and the emerging spheroid 3D models. Of concern, 3D cancer models are more cumbersome to grow, study, and evaluate for therapy response [12]. Since the first publication on cancer spheroids in 1971 [13], 3D culturing comprise a mere 1.7% of publications indexed in PubMed using cell lines in cancer research. Hence, there is a void of data from 3D models, and even more so for comparative experiments between 2D and 3D cultures.

Whereas cancer spheroid metabolism has largely been studied using omics [14, 15] and tracing [16] approaches, assessment of metabolic flux in cancer spheroids remains limited [17, 18]. Metabolic flux assessments can be performed in vitro by using the Seahorse XF Analyzers to measure how cells tolerate stressors such as drugs or toxins by altering their metabolism.

To better understand how 3D spheroid growth and metabolism differ from corresponding 2D-grown cells, we performed parallel studies evaluating a range of metabolic flux parameters and related metabolic protein markers in the colon cancer cell lines SW948 and HCT116 and the pancreatic cancer cell lines Panc1 and MIA-Pa-Ca-2. To maintain the 3D cell cultures close to the glucose levels found in vivo [19], all experiments were performed using 5 mmol/L glucose in growth medium. In comparison, most cell culture media contain 25 mmol/L glucose. In the presented study, we show how growth rates, live metabolic flux measurements, substrate utilization, and related metabolic biomarkers correlate in corresponding 2D and 3D cell models.

## Methods

### Cell culture

Cell lines were chosen based on published results on metabolism [20, 21], and spheroid production [22–29]. The goal was to achieve some metabolic variation among the cell lines, so lines were chosen that were purported to differ in their dependence on glycolysis and oxidative phosphorylation. Since 3D culture was essential to this study, only cell lines that had already been confirmed to produce spheroids were considered. This led to the selection of SW948 and HCT116, colorectal cancer (CRC) lines, and Panc1 and MIA-Pa-Ca-2, pancreatic ductal adenocarcinoma (PDAC) cell lines.

SW948 and MIA Pa-Ca-2 were purchased from European Collection of Authenticated Cell Cultures (ECACC), Panc1 and HCT116 cell lines were generously provided by collaborators at the Stavanger University Hospital Molecular Biology Lab. All cell lines were cultured in DMEM (Corning, Corning, USA) supplemented with 10% fetal bovine serum (FBS) (BioWest, Nuaillé, France), 5 mM glucose (Sigma-Aldrich, St. Louis, USA), 2 mM l-glutamine (Corning, Corning, USA), penicillin (100 U/ml), streptomycin (100 μg/ml) (Merck Millipore Corporation, Burlington, USA) in a humidified incubator at 37 °C with 5% CO_2_ infusion. Cells were grown in 2D adherent culture conditions, from which spheroids were prepared before each experiment. Spheroids were formed from a 40-μl volume of detached single-cell suspensions with 5000 cells, either in hanging drops in a dish, or in CELLSTAR cell repellent U-bottom plates (Greiner Bio-One, Kremsmünster, Austria). Spheroids were grown for 3 days (CRC) or 4 days (PDAC) before conducting metabolic assays.

#### 2D doubling time

Cells were seeded in flat-bottom 96-well plate at a density of 5000 cells/well. At each timepoint, 3 wells were stained with Hoechst and the entire well imaged using Leica SP8 confocal microscope (Leica Microsystems, Mannheim, Germany) for direct cell detection and counting in the LASX software.

#### Spheroid growth

Spheroids were cultured in U-bottom plates as described above, but in densities from 200 cells/well to 10,000 cells/well. Transmitted light images of the spheroids were captured on days 3, 4, 6, and 8 using the Leica SP8 confocal microscope. Images were analyzed in ImageJ to obtain the cross-sectional area of the spheroid.

### Metabolic flux assays

Mitochondrial respiration and glycolysis were measured using the Seahorse XF96e and XFp flux analyzers (Agilent). For mitochondrial oxygen measurements, assay media consisted of unbuffered, serum-free DMEM 8.3 g/L (D5030, Sigma-Aldrich, St. Louis, USA), NaCl 1.85 g/L, 2 mM l-glutamine, and 5 mM glucose adjusted pH to 7.4 before running the experiment. Before running mitochondrial and glycolysis assays, titration of CCCP over a range of concentrations was performed as per the Seahorse cell characterization procedure. The CCCP concentration chosen was that which yielded the maximum OCR value, for each cell line and culture method (Table S2).

#### 2D assays

One day prior to assay analysis, 10,000 cells were seeded in each well of a XF96e cell culture plate using culture media as described above. Approximately 1 h before the assays, culture media was exchanged for 180 μl assay media. The plates were then incubated at 37 °C in a CO_2_-free incubator for 45 min–1 h prior to running the assay. Oxygen consumption rate (OCR) and extracellular acidification rate (ECAR) were measured over 90 min (15 mix and measure cycles), with compounds being injected every 3 cycles. For the mitochondrial respiration assays, the following compounds were injected sequentially (final concentrations in the wells): oligomycin (3 μM), CCCP (0.5 μM), rotenone (1 μM), and antimycin A (AMA, 1 μM) (all compound reagents from Sigma-Aldrich, St. Louis, USA). For the glycolysis assays, the assay media was not supplemented with glucose and the following compounds were injected sequentially (final concentrations in the wells): glucose (10 mM), oligomycin (3 μM), and 2-deoxy-D-glucose (100 mM). Protein concentration was measured in each well for normalization using standard BCA assay (PanReac AppliChem, Darmstadt, Germany) according to manufacturer’s instructions.

#### 3D assays

Spheroid cell culture plates were used for the XF96e assays. Each well was coated with 25 μl CellTak (Corning, Corning, USA) at a concentration of 33 μg/ml. Spheroids were transferred from hanging drops and placed in the centers of wells in the spheroid assay, which contained 160 μl assay media. Plates were then incubated at 37 °C in a CO_2_-free incubator for 45 min–1 h prior to running the assay. OCR and ECAR were measured over 150 min (23 mix and measure cycles). For the mitochondrial respiration assays, the following compounds were injected sequentially (final concentrations in the wells): oligomycin after cycle 3 (3 μM), CCCP after cycle 9 (cell line dependent, see Table S2), rotenone after cycle 15 (1 μM), and antimycin A after cycle 19 (1 μM). For the glycolysis assays, the assay media consisted of unbuffered, serum-free DMEM 8.3 g/L, NaCl 1.85 g/L, 2 mM l-glutamine, adjusted pH to 7.4 before running the experiment and the following compounds were injected sequentially (final concentrations in the wells): glucose after cycle 3 (10 mM), oligomycin after cycle 9 (3 μM), and 2-deoxy-D-glucose after cycle 15 (100 mM). To use the data for comparison of metabolic phenotypes across cell models regardless of absolute metabolic activity, OCR and ECAR were normalized to basal OCR levels (MST) and ECAR level after glucose injection (GST). Statistical significance was determined in GraphPad using ordinary one-way ANOVA, correction for multiple comparisons by the Sidak method, with alpha = 0.05.

### ATP production calculations

Buffer Factor of assay media was calculated using the Agilent Seahorse XF Buffer Factor Protocol. This number (1.70 mM/pH) was then used for the calculation of the ATP production rate after each injection following the information contained in the Agilent White Paper: “Quantifying Cellular ATP Production Rate Using Agilent Seahorse XF Technology” and Mookerjee et al .[30], according to the following equations:

1 $${\mathrm{PPR}}_{\mathrm{total}}=\mathrm{ECAR}/\mathrm{BP}$$

2 $${\mathrm{PPR}}_{\mathrm{resp}}=\left({10}^{\mathrm{pH}-{\mathrm{pK}}_{\mathrm{CO}2\to \mathrm{HCO}3}}/\left(1+{10}^{\mathrm{pH}-{\mathrm{pK}}_{\mathrm{CO}2\to \mathrm{HCO}3}}\right)\right)\bullet {\mathrm{OCR}}_{\mathrm{mito}}$$

3 $${\mathrm{PPR}}_{\mathrm{glyc}}={\mathrm{PPR}}_{\mathrm{total}}-{\mathrm{PPR}}_{\mathrm{resp}}$$

4 $${\mathrm{ATP}}_{\mathrm{glyc}}=\left[{\mathrm{PPR}}_{\mathrm{glyc}}\bullet \mathrm{ATP}/\mathrm{lactate}\right]+\left[{\mathrm{OCR}}_{\mathrm{mito}}\cdot 2\mathrm{P}/\mathrm{O}\right]$$

5 $${\mathrm{ATP}}_{\mathrm{ox}}=\left[{\mathrm{OCR}}_{\mathrm{coupled}}\bullet 2\mathrm{P}/\mathrm{O}\right]+\left[{\mathrm{OCR}}_{\mathrm{mito}}\cdot 2\mathrm{P}/\mathrm{O}\right]$$

6 $${\mathrm{ATP}}_{\mathrm{total}}={\mathrm{ATP}}_{\mathrm{glyc}}+{\mathrm{ATP}}_{\mathrm{ox}}$$

The buffer factor was converted to a buffering power (BP) of 0.258 mpH/pmol H+ and combined with data from the glycolysis assays and residual OCR after AMA from mitochondrial stress test, run in parallel to GST. The total proton production rate (PPR) is found by dividing ECAR by BP (Eq. 1) and can be broken down into PPR_resp_ and PPR_glyc_, with the PPR_resp_ being from all mitochondrial oxygen consumption (non-mitochondrial is subtracted) (Eq. 2) where pH = 7.4 and pK_CO2→HCO3_ = 6.093. PPR_glyc_ is the PPR_total_ minus PPR_resp_ (Eq. 3). The PPR is converted to ATP by calculations using known values for mol ATP yielded per mol oxygen consumed (P/O ratio). The result is ATP_glyc_ (Eq. 4) that incorporates all ATP produced through glycolysis, including that which ends in lactate production or pyruvate that is shuttled to mitochondria resulting in production of CO^2^ (conversion of CO^2^ to bicarbonate is a major source of extracellular acidification in Seahorse assays [31] due to the use of unbuffered media). In the last case, this may or may not be coupled to ATP production via OXPHOS. Metabolic phenotypes are described using the bioenergetic indices also described in Mookerjee et al. [30].

7 $$\mathrm{GI}=100\ \left({\mathrm{ATP}}_{\mathrm{glyc}}/{\mathrm{ATP}}_{\mathrm{total}}\right)$$

8 $$\mathrm{CI}={\mathrm{GI}\mathrm{g}}_{\mathrm{lucose}}-{\mathrm{GI}}_{\mathrm{basal}}$$

9 $$\mathrm{PI}={\mathrm{GI}}_{\mathrm{oligo}}-{\mathrm{GI}}_{\mathrm{glucose}}$$

The Glycolytic Index (GI) is a way to normalize between samples, allowing comparison of phenotype and contribution of glycolysis to ATP production regardless of changes in absolute ATP production rates (Eq. 7). The Crabtree Index (CI) quantifies the shift away from OXPHOS upon addition of glucose (Eq. 8) and The Pasteur Index (PI) quantifies the shift to OXPHOS upon “removal” of mitochondrial inhibitor oligomycin (Eq. 9). Statistical significance was determined in GraphPad using multiple *t* tests, correction for multiple comparisons by the Holm-Sidak method, with alpha = 0.05.

### Metabolite assays

Media was collected from spheroids grown in ULA round-bottom 96-well plates and 2D cultures from flat 96-well plates, at days 0, 2, and 4, with no refeeding of media during this period. Day 0 for spheroids is culture day 3 for CRC spheroids and day 4 for PDAC spheroids and on this day they received full media exchange. The media from each timepoint was then used in the metabolite assays. Glucose concentration was assayed using the GlucCell glucose monitoring system (Cesco Bioengineering, Taichungy, Taiwan) according to the manufacturer’s instructions. Lactate was assayed using the l-Lactate Assay Kit (MAK329, Sigma-Aldrich, St. Louis, USA) according to the manufacturer’s instructions. Glutamine was assayed using the Glutamine/Glutamate Determination Kit (GLN1, Sigma-Aldrich, St. Louis, USA), adjusted for a low volume assay in a microplate. These results include both glutamine and endogenous glutamate. All values are presented normalized to surface area of cultures grown for equivalent period as that in the. Surface area in 2D was obtained from area measured in images on day 4 from the proliferation experiments. Surface area in 3D was estimated from area of spheroids in growth experiments after the same time in culture and extrapolated to the surface area of sphere, *A = 4·π·r*^*2*^.

### Protein expression

Expression of metabolic proteins (MCT1, MCT4, GLUT1, UCP2, TOMM20) was measured by flow cytometry. Spheroids (approximately *n* = 600) comprised of 10,000 cells were produced and grown for 3–4 days before collection and dissociation using Accutase (Innovative Cell Technologies, Inc., San Diego). The spheroids were collected by rinsing plates with PBS and placed in a centrifuge tube. After centrifuging 5 min at 100 RCF, the supernatant was discarded and the pellet was resuspended in 5 ml Accutase. The tubes were placed on a rocker at room temperature and resuspended every 10 min using a P1000 pipet to gently disrupt the spheroids. Adherent cells were collected on the same day as the spheroids, also detached using Accutase. The cell lines varied considerably in incubation time needed for complete detachment and dissociation. Cell suspensions were counted using a Muse cell analyzer and Muse count and viability assay (Luminex, Austin, USA). The cell suspensions were then fixed with 3.7% PFA for 30 min. Cells were kept in PBS until staining. 5 × 10^5^ cells were used for each staining reaction (at 1 × 10^6^ cells/ml). Each tube was incubated in blocking/permeabilization buffer (PBS, 20% FCS, 0.05% Tween 20) for 1 h at room temp, rinsed with PBS before adding primary antibodies (single stain per tube) and incubated overnight at 4 °C, rocking. The next day, they were rinsed with PBS and the secondary antibodies were added, with the exception of unstained control and the tubes stained with conjugated TOMM20 antibody. Finally, cells were rinsed and resuspended in PBS containing 0.5% BSA for analysis in Bio-Rad S3e (Bio-Rad Laboratories, Hercules, USA) (experimental replicates 1 and 2) and CytoFlex (Beckman Coulter, Brea, USA) (experimental replicate 3). Primary antibodies: Rabbit Anti-Glucose Transporter GLUT1 antibody, EPR3915 (Abcam, Amsterdam, Netherlands); Mouse MCT1 Antibody SC-365501 and Mouse MCT4 Antibody SC-376140 (Santa Cruz Biotechnology, Dallas, USA); Rabbit UCP2 antibody (Bioss Antibodies Inc., Woburn, USA); Llama anti-rabbit IgG polyclonal antibody, CF™ 488A and anti-mouse IgG polyclonal antibody, CF™ 488A (Biotium, Fremont, USA). Datasets were analyzed using FCS Express (De Novo Software, Pasadena, USA), gated to singlet populations, and the median values were compared in ratios of 3D to 2D.

## Results

### Characterization of cell lines in 2D and 3D culture

**Fig. 1:**
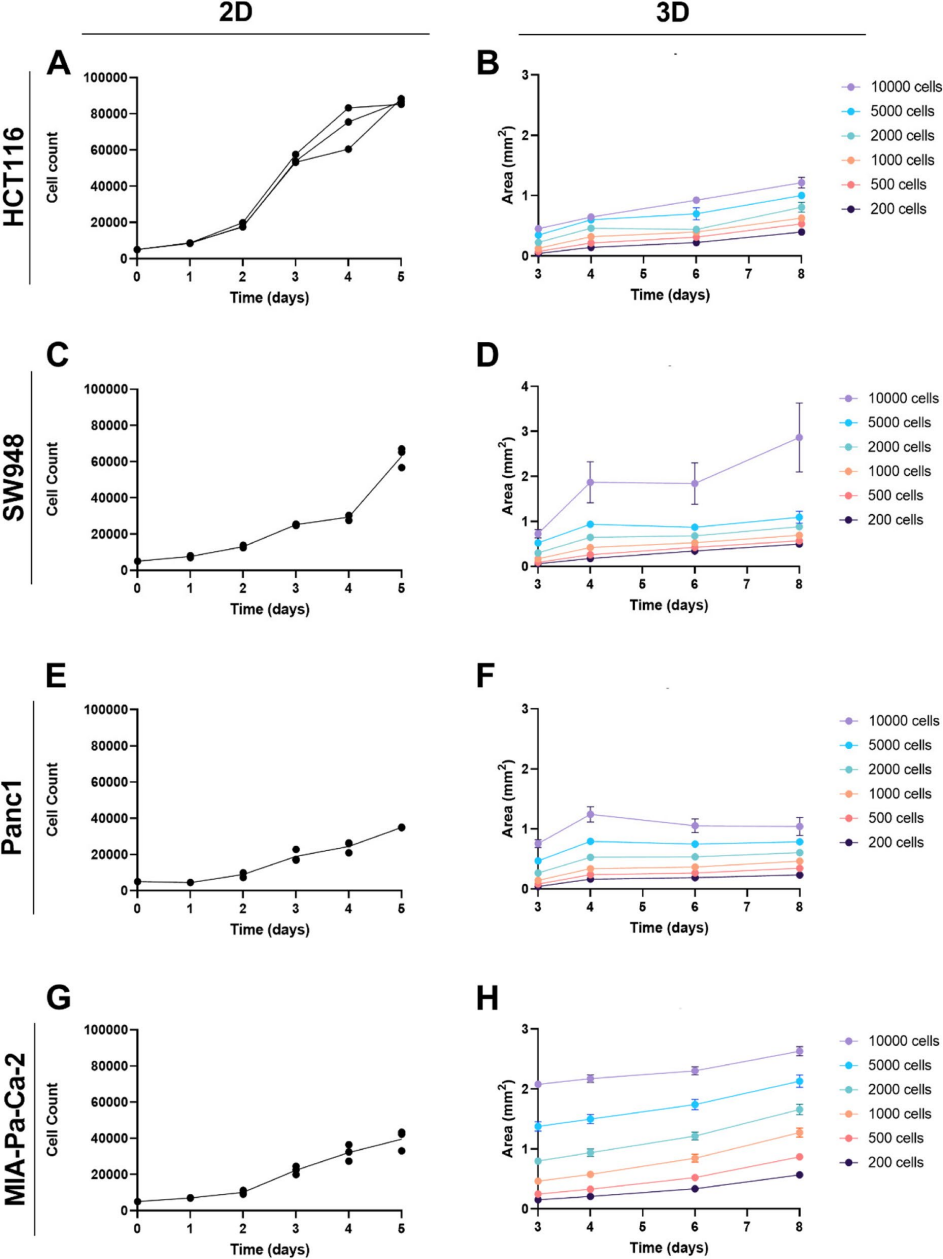
Cell line proliferation in 2D and spheroid growth. Proliferation of cells in 2D was measured by direct cell counting in a flat-bottom 96-well plate over a period of 5 days, starting cell number of 5000 cells (*n* = 3 wells): HCT116 (**A**), SW948 (**C**), Panc1 (**E**), MIA-Pa-Ca-2 (**G**). For spheroid growth measurements, 5000 cells were seeded in different amounts in ultra-low-attachment round bottom plate and then imaged from days 3-8 (*n* = 10 spheroids, 1 per well): HCT116 (**B**), SW948 (**D**), Panc1 (**F**), MIA-Pa-Ca-2 (**H**). Measured area is plotted and error bars indicate standard deviation

Cell proliferation rate and cell doubling time are affected by cell growth conditions. To determine the cell doubling time of each cell line used when grown in physiological glucose conditions, direct cell counting using Hoechst staining was performed over a period of 5 days (Fig. 1, left column). HCT116 has the shortest doubling time of 17.9 h (Fig. 1A), compared to the other colorectal cell line, SW948, which was 10 h longer with 27.9 h doubling time (Fig. 1C). For the pancreatic cell lines, the doubling time of Panc1 is 24.8 h (Fig. 1E), versus 28.8 h for MIA-Pa-Ca-2 (Fig. 1G).

Spheroids were formed from each cell line, using cell numbers ranging from 200 to 10,000 cells, and growth was monitored during 8 days (Fig. 1B, D, F, H). Representative images of the 3D spheroids are shown in supplementary data (Figure S1). The initial size measurement was performed on day 3 after 3D spheroid formation. The colorectal cancer cell line HCT116 forms spheroids that are compact in size and where cells are indistinguishable from another (Figure S1). The size of spheroids ranged from 0.04 mm^2^ in cross-sectional area formed from 200 cells to 0.45 mm^2^ from spheroids formed by 10,000 cells (Fig. 1B). Over the following 5 days of measurement the lowest cell density of 200 cells increased 10-fold in area to 0.40 mm^2^, whereas the highest cell density of 10 000 cells increased only 3-fold to 1.21 mm^2^ from the initial measurement. The other colorectal cancer cell line, SW948 also form compact spheroids of similar size as HCT116 starting from 0.06 mm^2^ from 200 cells, to 0.73 mm^2^ formed from 10,000 cells. The spheroid growth for the lowest cell density increases by 8-fold (0.49 mm^2^) over the 5 days whereas the highest cell density is nearly quadruples in size to 2.87 mm^2^ in the same time period (Fig. 1D). HCT116 spheroids are more spherical and vary less in size than SW948, based on visual inspection and standard deviation of measurements. The CRC spheroids start developing a dark necrotic core by day 3 which is more pronounced in SW948, and have a tendency to fracture and lose their structural integrity once they reach larger sizes (Figure S2).

Panc1 forms spheroids with a clear edge and single cells remain distinguishable (Figure S1). Spheroids range from 0.04 mm^2^ (200 cells) to 0.76 mm^2^ (10,000 cells) in area and grow to 0.23 mm^2^ (200 cells) and 1.04 mm^2^ (10,000 cells). MIA-Pa-Ca-2 form large spheroids that are easily dissociated and with an irregular shape (Figure S1). These spheroids are in general larger in size ranging from 0.15 mm^2^ (200 cells) to 2.08 mm^2^ (10,000 cells), and exhibit less growth compared to the other cell lines over the 8 days of size measurements (Fig. 1H). Panc1 develops a dark core and at a later time point than seen for the CRC cell lines (day 6 in 5000-cell spheroids), while this darker core is not detected in MIA-Pa-Ca-2 over the 8 days of size measurements (Figure S2). 2D characteristics and cell appearance were not predictive of 3D spheroid morphology (Figure S1). Nor was there an apparent relationship between doubling time in 2D and size or growth in 3D.

### Basal ATP-linked respiration is increased in 3D cultures, compared to 2D

**Fig. 2:**
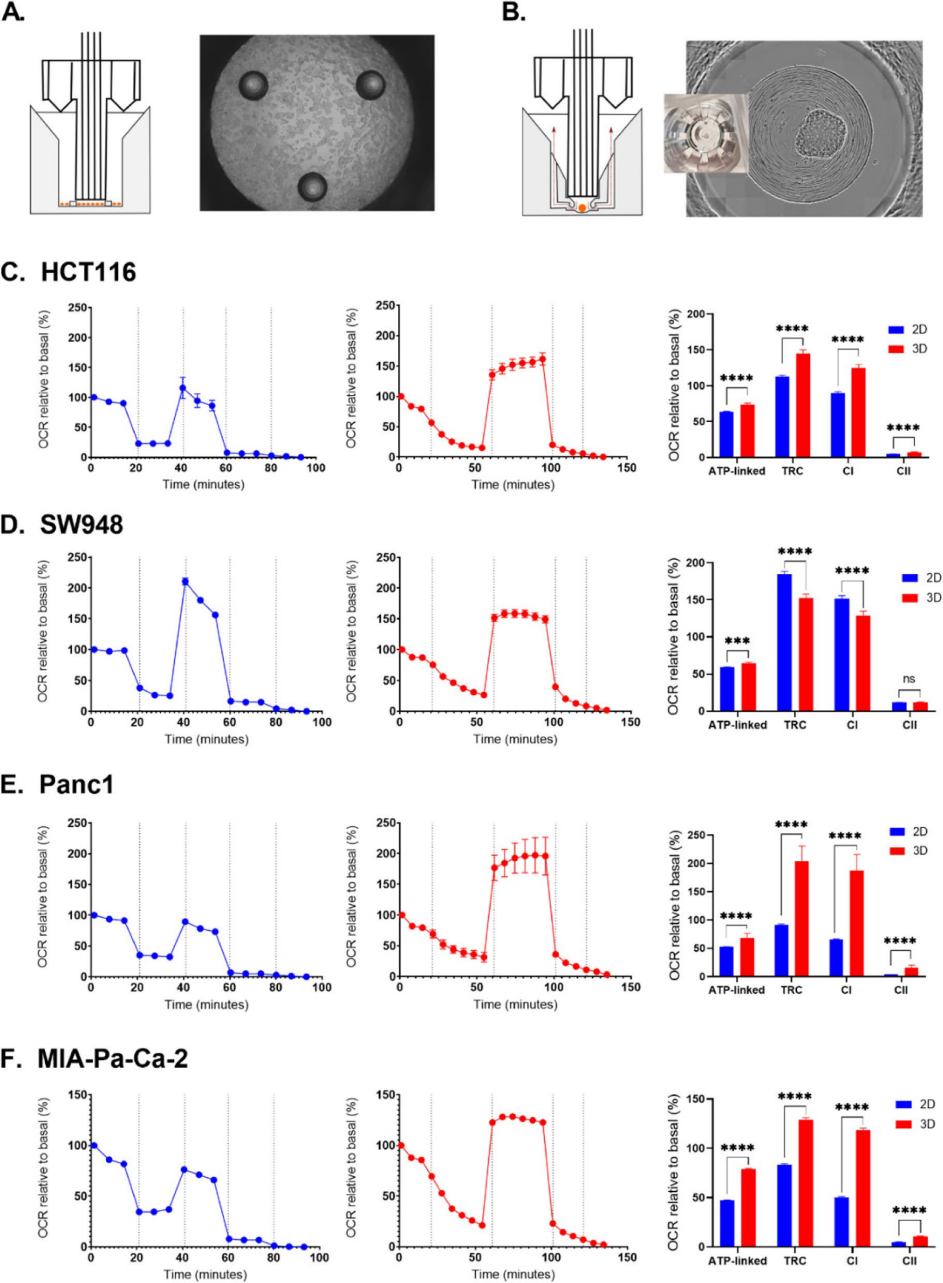
Results from Mitochondrial Stress Test assays in 2D and 3D by cell line. **A** Seahorse set-up for 2D analysis. **B** Seahorse set-up for 3D analysis. Mitochondrial Stress Test (MST) traces (left and middle rows) present OCR data normalized to the maximum basal value of each sample. Dotted vertical lines indicate injections in the following order: oligomycin, CCCP, rotenone, antimycin A. Metrics from the MST (right row) include ATP-linked respiration (OCR basal max–oligomycin min), total respiratory capacity (maximum OCR after CCCP), complex I activity (OCR CCCP–rotenone) and complex II activity (OCR rotenone–antimycin A). 2D results are in blue and 3D results are in red. Error bars represent SEM. **C** HCT116, 2D *n* = 56 and 3D *n* = 38. **D** SW948, 2D *n* = 60 and 3D *n* = 37. **E** Panc1, 2D *n* = 57 and 3D *n* = 4. **F** MIA-Pa-Ca-2, 2D *n* = 54 and 3D *n* = 47. Statistical significance was determined using multiple *t* tests, correction for multiple comparisons by the Holm-Sidak method, with alpha = .05. *p* < 0.05: *, *p* < 0.001: **, *p* < 0.0001: ***, *p* < 0.00001: ****

**Fig. 3:**
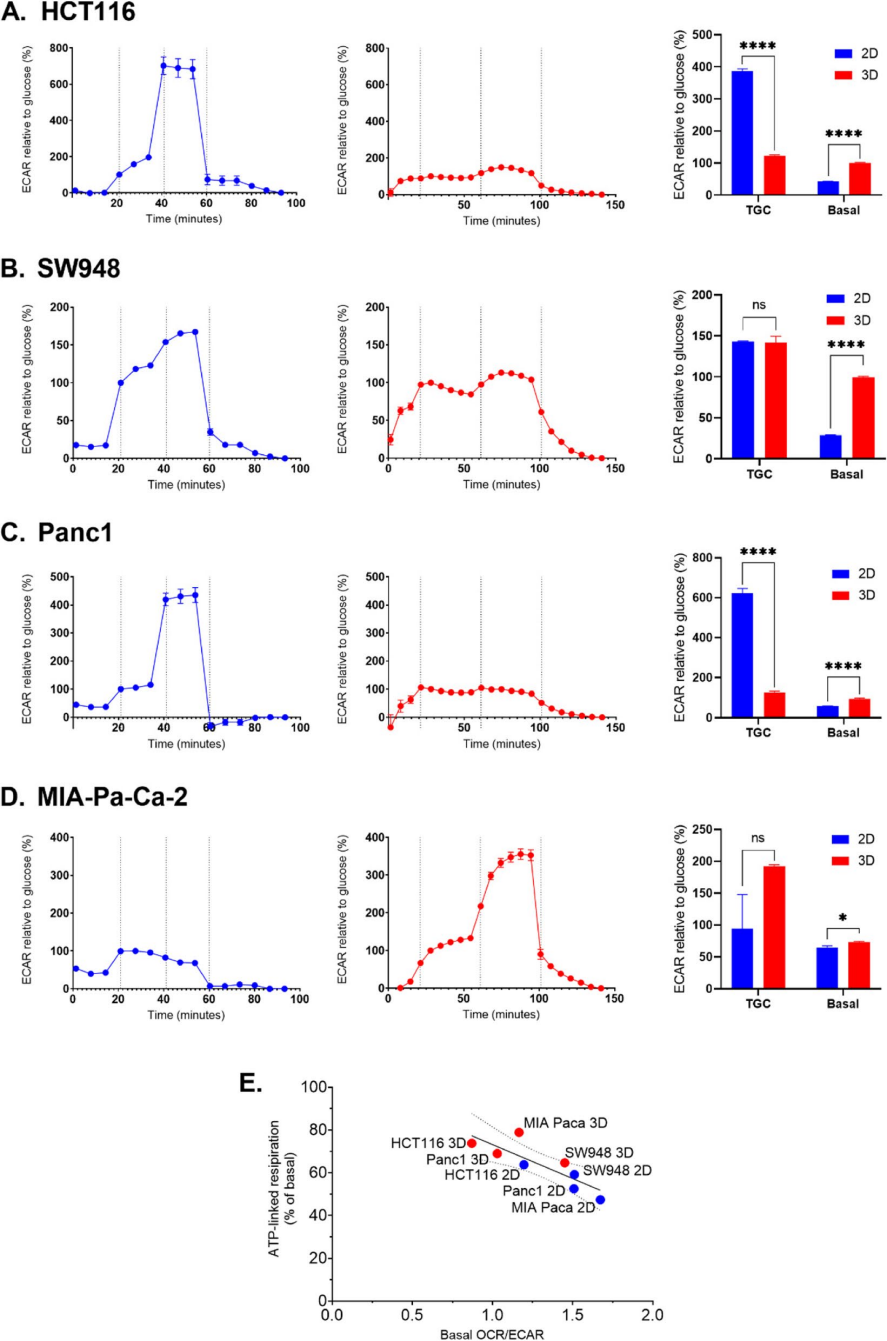
Results from Glycolysis Stress Test assays in 2D and 3D by cell line. Glycolysis Stress Test (GST) traces (left and middle rows) present ECAR data normalized to the ECAR of each sample after glucose injection. Dotted vertical lines indicate injections in the following order: glucose, oligomycin, 2-DG. Metrics from the GST (right row) include basal ECAR without glucose and total glycolytic capacity (maximum ECAR after oligomycin). 2D results are in blue and 3D results are in red. Error bars represent SEM. **A** HCT116, 2D *n* = 57 and 3D *n* = 25. **B** SW948, 2D *n* = 55 and 3D *n* = 47. **C** Panc1, 2D *n* = 54 and 3D *n* = 10. **D** MIA-Pa-Ca-2, 2D *n* = 25 and 3D *n* = 46. Statistical significance was determined using multiple *t* tests, correction for multiple comparisons by the Holm-Sidak method, with alpha = 0.05. *p* < 0.05: *, *p* < 0.001: **, *p* < 0.0001: ***, *p* < 0.00001: ****. **E** ATP-linked respiration (% of basal respiration) vs basal OCR/ECAR, with 3D in red and 3D in blue. Simple linear regression, *R*^2^ = 0.7004, with dotted bands showing 95% confidence interval. Pearson correlation = − 0.837, *p* = 0.0096

Real-time metabolic flux was assessed in 2D and 3D using the Seahorse XFe96 system (Fig. 2A, B). OCR and ECAR from the Mitochondrial Stress Test (MST) and Glycolysis Stress Test (GST) were used to calculate relevant metabolic metrics for more information on the phenotypes of the cell lines in 2D and 3D, relative to either basal OCR (MST, Fig. 2) or ECAR after glucose injection (GST, Fig. 3). The spheroids were also assayed using the Seahorse XFp system (Figure S3) and MST. Protein normalization was performed for OCR and ECAR values in 2D and 3D (Figure S4).

In HCT116 (Fig. 2C), the respiration-linked ATP production increases in spheroids, together with an increase in total respiratory capacity compared to 2D-grown cells. There is also a slight increase in complex II activity in spheroids versus 2D cells, as measured after rotenone inhibition. Comparatively, HCT116 spheroids have significantly lower reserve glycolytic capacity (287% vs 23%) in spheroids, and higher basal glycolysis (43% vs 100%) (Fig. 3A). In SW948 spheroids, ATP-linked respiration is significantly higher, 5.5 percentage points (pp) (*p* < 0.0001), but respiratory capacity is 32 pp lower, *p* < 0.00001 (Fig. 2D). There is no significant change in glycolytic reserve capacity, but basal glycolysis increases from 29 to 99% in spheroids (*p* < 0.00001) (Fig. 3B).

Panc1 spheroids increase in respiratory metrics but this change (16.5 pp) is only significant for ATP-linked respiration, *p* < 0.05 (Fig. 2E). Like HCT116, glycolytic reserve capacity drops significantly in spheroids compared to 2D, *p* < 0.00001 (Fig. 3C) from 521% to 21%. With this, basal glycolysis increases from 58 to 95%, *p* < 0.00001. MIA-Pa-Ca-2 spheroids have significantly higher respiratory metrics, *p* < 0.00001 (Fig. 2F). ATP-linked respiration increases 34pp, respiratory capacity increases 46 pp, and complex II activity increases 6 pp. Reserve glycolytic capacity increases in MIA-Pa-Ca-2 spheroids (Fig. 3D), but not significantly as there is a high amount of variation between the individual samples. Basal glycolysis increases significantly (8 pp, *p* < 0.05).

Except for SW948, the other cell lines all trend to higher respiratory activity in 3D culture (increase in ATP-linked respiration, respiratory capacity, and complex II activity). ATP-linked respiration in 3D is significantly higher in all cell lines. Changes in total glycolytic capacity between 2D and 3D are more varied. HCT116 and Panc1 exhibit changes in the same direction for both respiration and glycolysis, but not on the same scale. However, the 3D models of all the cell lines demonstrate lower OCR/ECAR than their 2D counterparts, reflecting a more glycolytic phenotype. When combining 2D and 3D samples, ATP-linked respiration is negatively correlated to basal OCR/ECAR (Fig. 2E) (− 0.837, *p* = 0.0096), so the lower the OCR/ECAR, the more respiration is linked to ATP production.

### Spheroids depend on glycolysis for the majority of ATP production

**Fig. 4:**
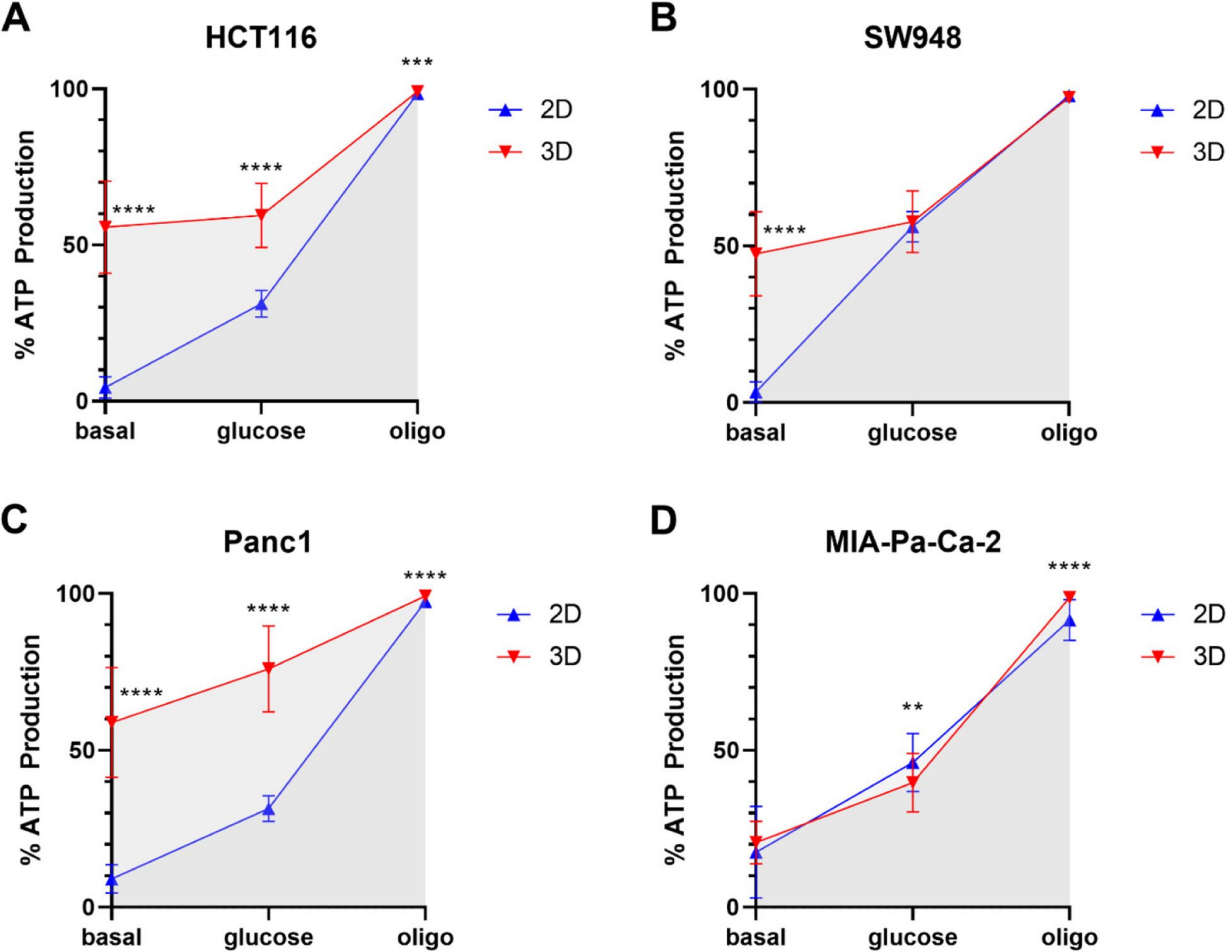
ATP production from glycolysis. Percentage of total ATP produced that is attributed to glycolysis was calculated using metrics from Seahorse assays in 2D (blue) and 3D (red) over 2 independent experiments: **A** HCT116, 2D *n* = 57 and 3D *n* = 25. **B** SW948, 2D *n* = 55 and 3D *n* = 47. **C** Panc1, 2D *n* = 54 and 3D *n* = 10. **D** MIA-Pa-Ca-2, 2D *n* = 51 and 3D *n* = 46. Basal is without glucose, glucose is after the addition of glucose, and oligo is after oligomycin injection. Points represent the mean value over all wells, error bars are standard deviation. Statistical significance was calculated by unpaired *t* test, multiple comparisons (two-stage linear step-up procedure of Benjamin, Krieger and Yekutieli, FDR 1%): *p* < 0.05: *, *p* < 0.001: **, *p* < 0.0001: ***, *p* < 0.00001: ****

Percent of ATP production from glycolysis and oxidative phosphorylation (OXPHOS) was calculated using results from the Seahorse assays (Eqs. 1–6), as described in Mookerjee et al. [30]. Table 1 and Fig. 4 show the calculated percent non-aerobic ATP production (Eq. 7) in 2D and 3D cultures at measurements with no glucose added (basal), after glucose injection (glucose), and after oligomycin injection (oligo). The data generated from this assay allows the quantification of bioenergetic phenotypes of the cell models [30]. The Crabtree Index (CI) quantifies the shift away from OXPHOS upon addition of glucose (Eq. 8). The Pasteur Index (PI) describes the shift to OXPHOS upon ‘removal’ of ATP-synthase inhibitor oligomycin (Eq. 9). This reflects the flexibility of the cells to shift their metabolism to glycolytic from oxidative pathways depending on acute changes in the availability of glucose and oxygen. These phenotypes are not to be compared to the Warburg effect, which is a description of a chronic metabolic phenotype, and is best represented by just the glycolytic index (GI) in the presence of glucose, where a chronic Warburg phenotype would be that over 50%. HCT116 spheroids are significantly more glycolytic in all measurements of the assay, *p* < 0.00001 (basal and glucose) and *p* < 0.0001 (oligo) (Fig. 4A). SW948 (Fig. 4B) only differ between 3D and 2D at the basal stage. After glucose and oligomycin, SW948 use glycolysis for ATP production at about the same percentage. Like HCT116, Panc1 spheroids are significantly more glycolytic at every stage of the assay, *p* < 0.00001 (Fig. 4C). In contrast to the other cell lines, MIA-Pa-Ca-2 are more glycolytic in 2D after glucose injection, but at basal, they do not differ significantly (Fig. 4D). However, after oligomycin, MIA-Pa-Ca-2 are significantly more glycolytic in 3D. The level of ATP production from glycolysis after oligomycin in 2D MIA-Pa-Ca-2 culture stands out as the lowest of all the cell lines; any activity remaining after oligomycin is due to substrate-linked phosphorylation from the TCA cycle not linked to OXPHOS. The spheroids all exhibit a lower CI, lower PI, and higher GI_glucose_ compared to 2D, with the exception of MIA-Pa-Ca-2 which have higher PI and GI_glucose_ in 3D.

**Table 1:** ATP Production (%) from glycolysis

Cell line	Method	GI_**basal**_	CI	GI_**glucose**_	PI	GI_**oligo**_
HCT116	2D	4.4 ± 3.4%	26.8	31.2 ± 4.3%	67.2	98.4 ± 0.2%
	3D	55.7 ± 14.8%	1.7	59.4 ± 10.2%	29.7	99.1 ± 1.2%
SW948	2D	3.4 ± 3.3%	52.7	56.1 ± 4.9%	41.8	97.9 ± 0.6%
	3D	47.4 ± 13.5%	10.3	57.7 ± 9.9%	39.7	97.4 ± 1.6%
Panc1	2D	9.0 ± 4.5%	22.5	31.5 ± 4.1%	65.9	97.4 ± 0.5%
	3D	58.9 ± 17.5%	17.0	75.9 ± 13.7%	23.3	99.2 ± 0.6%
MIA-Pa-Ca-2	2D	17.5 ± 14.6%	28.7	46.2 ± 9.3%	45.3	91.5 ± 6.5%
	3D	20.7 ± 6.8	19.0	39.7 ± 9.4%	59.0	98.7 ± 0.8%

Glycolytic Index (GI) and bioenergetic phenotypes calculated from Seahorse assay data [30]. GI_basal_ is before supplementation with glucose, GI_glucose_ is after supplementation with glucose, and GI_oligo_ is after injection with oligomycin. Crabtree Index (CI) = GI_glucose_ – GI_basal_. Pasteur Index (PI) = GI_oligo_ – GI_glucose_

### Differential expression of proteins involved in metabolism in 2D versus 3D cultures

**Fig. 5:**
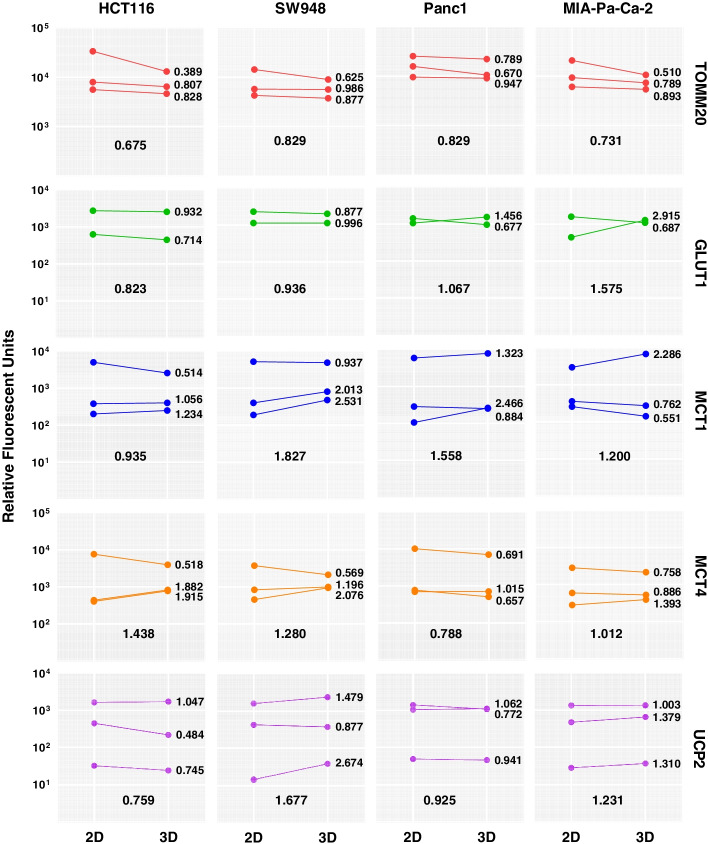
Expression of metabolic proteins by flow cytometry. Each panel shows the median fluorescent intensity of detected proteins in dissociated single cells from 2D and 3D cultures, by cell line (columns) and protein (rows). A line connects each matched 2D and 3D experimental replicate and fold difference in intensity (3D/2D) is shown to the right of each line. Average fold difference over all experiments is at the bottom of each panel

The expression levels of relevant metabolic proteins were analyzed using flow cytometry to investigate their potential role as markers to support or explain the results of the metabolic flux assays. This includes the major membrane proteins involved in nutrient transport represented by glucose transporter 1 (GLUT1), monocarboxylate transporter 1 (MCT1), and monocarboxylate transporter 4 (MCT4) and two mitochondria-associated proteins, translocase of outer mitochondrial membrane 20 (TOMM20) and uncoupling protein 2 (UCP2). Median protein expression is plotted in Fig. 5 and fold difference of 3D over 2D is added alongside each experimental result (histograms for each individual population can be found in Figure S5). While there is some inter-run variation, on average a large increase in MCT transporters (MCT1 and/or MCT4) is seen in CRC spheroids, with 1.438 fold difference in HCT116 in MCT4 and 1.827 (MCT1) and 1.280 (MCT4) fold difference in SW948. Panc1 spheroids demonstrate opposite expression level changes in these transporters, with higher expression in MCT1 (1.558) and lower expression in MCT4 (0.788). MCT1 expression varies over experimental replicates in MIA-Pa-Ca-2 although there is an average increase and MCT4 expression is on average unchanged between 2D and 3D. Little change is seen in GLUT1 in CRC while in PDAC, conflicting changes in expression are seen between runs. TOMM20 expression is lower across all cell lines in 3D culture. UCP2 increases in SW948 (1.677) and MIA-Pa-Ca-2 (1.231), while HCT116 exhibits a decrease in UCP2 (0.759). The forward scattering detected for each cell sample, an indication of cell size, differs between the 2D and 3D samples. The cells dissociated from the spheroids exhibit a lower median forward scattering and an increase in variation coefficient (except in HCT116), a reflection of greater distribution in spheroid cell size (Table S1).

### Substrate consumption is similar between 2D and 3D culture conditions

**Fig. 6:**
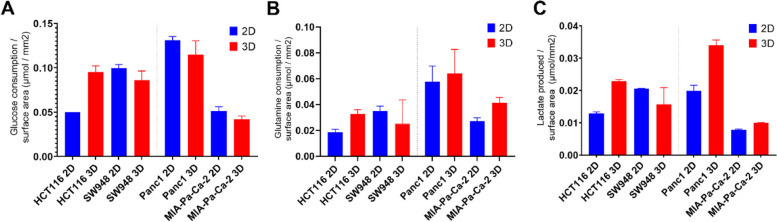
Glucose, lactate, glutamine levels in culture media from 2D and 3D cultures. Culture media was collected over a period of 4 days and levels of metabolites were measured in 2D (blue) and 3D (red) cultures and presented as mean level per media-exposed surface area (mm^2^). **A** Total glucose consumed, mean of 2 samples. **B** Total glutamine consumed (μmol), mean of 3 pooled samples. **C** Total lactate produced (μmol), mean 2 samples. Error bars represent standard deviation

All the experiments here have been carried out in low glucose as this is one way to achieve a more physiological cell culture environment. To ensure the cultures are not being starved of nutrients and for more insight into nutrient utilization, the levels of glucose, glutamine, and lactate in the culture media were tested (Fig. 6). All cell lines exhibit higher glucose consumption, lactate production, and glutamine consumption in 2D cultures when comparing absolute levels (Figure S6). However, when normalizing to surface area exposed to media, consistent differences disappear. HCT116 in 3D has almost double the amount of glucose consumed per mm^2^ than in 2D, but the other cell lines are quite similar between 2D and 3D cultures. The increase in HCT116 in 3D is also present for glutamine consumption and lactate production. MIA-Pa-Ca-2 in 3D consumes more glutamine per mm^2^ than in 2D and Panc1 in 3D produces almost twice as much lactate as in 2D. This is especially large considering there is no increase in glucose consumption in 3D. Finally, despite being cultured in physiological glucose, the cells are not being starved of glucose, even over 4 days without media exchange (Figure S6), with final concentrations never less than 1.1 mmol.

## Discussion

There are few published studies analyzing 3D spheroid metabolic flux. Metabolic phenotype data from literature is difficult to compare as nutrient conditions, experimental design, and metrics presented differ between studies. We find that glucose metabolism in 3D spheroids differs significantly from what is measured in 2D cultures, both in terms of glycolytic and oxidative phosphorylation metrics in both CRC and PDAC. Other papers publishing both metabolic flux data and spheroid experiments have largely just run metabolic analysis on the 2D cultures used to produce the spheroids [32]. Our data clearly indicate that these data are not transferrable between culture methods as the metabolic phenotype shift from 2D to 3D culture in one cell line is greater than the phenotypic differences between each cell line and tumor source.

We found that spheroids have higher non-aerobic ATP production in the absence of added glucose compared to 2D grown cells. The calculations of ATP production are based on assumptions that no other nutrients are provided and any ATP production without glucose is due to glycogen metabolism [30]. In our experiments, the cell growth media contained glutamine which also serves as a metabolic substrate, and thus complicates the simplified model presented by Mookerjee et al. [30]. However, this retained non-aerobic ATP production was not found in the 2D cultures grown in the same glutamine-enriched media. As the majority of glutamine consumed is metabolized via glutaminolysis to lactate [5] and therefore also contributes to ECAR, this may explain the continuous increase in ECAR from the 3D spheroids in glucose-free conditions. In low-oxygen conditions, as found in the core of large spheroids, glutamine also makes aerobic glycolysis more effective and can sustain cell growth even as the exclusive substrate [33]. In support of this, we found that there was a decrease in glutamine concentration in the cell media for our 2D and 3D grown cell lines, although the 3D spheroids seem to be better at exploiting this substrate for energy conversion in glucose-free conditions. Furthermore, glycogen is a possible substrate, as pre-supposed in the Mookerjee model. HCT116 spheroids have been shown to have much higher percentage of cells in arrested growth (G0/G1) and higher expression of glycogen enzymes than cells in 2D culture [34]; glycogen has been found at higher amounts in more quiescent cells [35] which could explain this potential connection. The high level of ECAR seen in the glucose-free condition was an unexpected result and warrants more investigation to pinpoint plausible causes.

Upon analyzing mitochondrial function, we found that spheroids had higher ATP-linked respiration than the corresponding 2D cultured cells. For standard 2D cell growth, HCT116 is generally known to be highly metabolically active and oxidative [21]. In contrast, we have previously found that SW948 is more glycolysis-dependent than oxidative in 2D [36]. The pancreatic cell lines Panc1 and MIA-Pa-Ca-2 have previously been found to be lipogenic/oxidative [21] and glycolytic, respectively [20, 37]. We do find that these relative phenotypes persist in our physiological glucose setting for our 2D grown cells, however in 3D all shift to a more glycolytic phenotype. As such, 2D metabolic analysis cannot substitute for 3D analysis, as agreed by others in field [18, 38, 39], and needs to be acknowledged when screening for response to metabolic drugs. Only a few peer-reviewed studies are published that have run Seahorse assays for metabolic flux analysis of spheroids [18, 40, 41]. One used HCT116, showing increased spare respiratory capacity over 2D [42], in agreement with our results in HCT116 and the two PDAC cell lines in this study. Other metabolic phenotype data from literature is difficult to compare as nutrient conditions, experimental design, and metrics presented are too diverse. Despite some variation due to these factors, some repeated findings in 3D metabolism studies include increased glycolytic activity compared to monolayer cultures [38, 39, 43], phenotypic heterogeneity [44], and utilization of other nutrient sources than glucose [16, 39]. Reduction in levels of ATP-synthase subunits in spheroids [38] could be related to the increased ATP-linked respiration and reduced sensitivity to oligomycin seen in the presented study and elsewhere [18]. The increase in ATP-linked respiration in more glycolytic phenotypes (lower OCR/ECAR) could be due to an increased need for ATP-production efficiency in the mitochondria to compensate any lost ATP production in the shift to ATP-inefficient glycolysis. Alternatively, and more in line with the changes seen in absolute values, the lower OCR/ECAR could be a reflection of a decrease in OXPHOS, increasing the relative amount of glycolysis, with the remaining respiration more efficiently linked to ATP.

Changes in metabolism between 2D and 3D extend beyond functional phenotypes, and several metabolic biomarkers can be assessed. We found that mitochondrial transport protein, TOMM20, expression decreases in all spheroids compared to 2D, and monocarboxylate transporter (MCT) expression increases in spheroids of HCT116, SW948, and Panc1 that differ significantly from 2D in basal glycolysis. The change in TOMM20 could be related to the increase in mitochondrial ATP-coupling as TOMM20 overexpression has shown to affect proliferation of colorectal cancer, impacting ATP production and mito-potential [45]. Furthermore, the expression could reflect the cell size; however, the dissociated cells from 3D are only marginally smaller on average and may not fully explain the great difference in TOMM20 expression, especially in HCT116 and MIA-Pa-Ca-2. Whereas the change in expression of MCT between 2D and 3D cultures is variable between the cell lines, the results are interesting from the perspective of intra-spheroid nutrient cooperation. The spheroid models that do exhibit a change in expression compared to 2D are those that form more “spherical” 3D spheroids (Figure S1). Compared to 2D, MCT1 in spheroids is increased in SW948 and PANC1, while MCT4 is increased in the CRC cell lines, HCT116 and SW948. MCT4 is associated with glycolytic metabolism [46] and oxidative stress [47], whereas MCT1 and TOMM20 are markers of tumor areas that are proliferative and mitochondria-rich [47]. MIA-Pa-Ca-2 does not show clear change in MCT expression; these results are more variable in the biological replicates, and this cell line produces very flat and easily dissociated spheroids. A dynamic expression of nutrient and waste transporters based on a complex interplay between neighboring cells have previously been shown upon investigation of the MCT expression in cancer associated fibroblasts and cancer cells [48]. This could explain why spheroids with low cell to cell attachment may affect the expression of these receptors by nutrient signaling [49].

Despite the significant shift to glycolysis in the spheroid cultures shown in the present study, the expression of GLUT1 did not show the same significant change between 2D and 3D cultures. Increased GLUT1 expression has been associated with cancer aggressiveness [50–52], and many studies point to the possibility of GLUT1 as a prognostic marker [53]. However, these studies typically compare expression between cancer and normal tissues. Upon comparison of different cancer cell line models in 2D and 3D cultures, we found that GLUT1 protein expression in vitro was highly variable between 2D and 3D and not consistent nor directly correlated to the metabolic profile of the cell lines. The changes in glycolysis and corresponding glucose demand between the models may not be as extreme as that between normal to cancer cells, thus not inducing a significant change in GLUT1 expression. However, Vyas et al. have found GLUT1 gene expression significantly increased in HCT116 spheroids compared to 2D culture [34]. GLUT1 is the ubiquitous glucose transporter [54] and has a high-affinity to glucose (1–2 mM) [55]. We have previously found that different glucose conditions significantly affect GLUT1 expression in 2D [36]. However, as none of these models reached a level of extracellular glucose below 1 mM, this is not expected to effect GLUT1 expression. GLUT3 has a high glucose affinity [55] and has also been identified as a potential prognostic marker in cancer survival [56]. It is worth consideration if it has alternative potential as a sensitive detector of intracellular glucose demand. Levels of both glucose and lactate are vital for the survival of cancer cells in three-dimensional settings; thus, the transporters controlling these are worth further investigation [39]. Despite the extracellular levels of glucose measured at 1 mM, we surmise that the glucose may be exhausted in the core of the spheroid due to natural gradients and diffusion, as expected in such a model [9–11]. This plus an assumed low oxygen partial pressure in the spheroid core may be driving necrosis [57] and related to the metabolic changes seen here.

This study is primarily limited by the few cell lines included. Studying more cell lines and including tumor-derived organoids can provide a more complete view of the variation among 3D models and correlation with markers. Other improvements include sectioning spheroids and performing immunohistochemistry for localization of protein expression [58, 59]. Another option is layered removal of spheroids [60] for flow analysis; however, the dissociation of the spheroids here before fixation could be the source of some of the variability in protein expression. Even though the dissociation reagent used is quite gentle, some spheroids incubate for long time periods and this could influence protein expression, especially for very dynamic markers such as UCP2 [61]. Finally, an enhanced model of spheroid metabolism would include more physiological culture medium [62, 63], co-culture, and embedding in a matrix, but a matrix can present further challenges for analysis. Additionally, carbon tracing of glucose offers the ability to directly monitor metabolic flux and how glucose is being processed through the cells. Finally, modulation of expression of the markers via knock-down would add insight to how they directly affect the metabolic phenotypes.

## Conclusions

We find that metabolism changes significantly when cells are cultured in 3D, compared to 2D. In our data, spheroids demonstrate an increase in glycolytic activity over monolayer cultures, and the complexity of the 3D culture environment allows for improved utilization of other nutrient sources than glucose for ATP production. Spheroids have higher ATP-linked respiration in standard nutrient conditions and higher non-aerobic ATP production in the absence of supplemented glucose. Mitochondrial transport protein, TOMM20, expression decreases in all spheroid models compared to 2D, and monocarboxylate transporter expression increases in 3 of the 4 spheroid models. To our knowledge, the presented study is the most robust analysis comparing 2D and 3D spheroid metabolism using live metabolic flux measurements. Our results show that investigation of cancer metabolism should focus on using more complex 3D in vitro cell models to expand our knowledge within this field and gain a better understanding of the applicability to tumor biology. Even more importantly, the recent surge in screening and repurposing established metabolic drugs for cancer treatment should be done in 3D cell models to improve the translation into in vivo tumor settings.

## Supplementary Information

**Additional file 1: Figure S1.** Morphology of cell lines. 2D adherent (top) and 3D spheroid (bottom) cultures. (Spheroid size for detail, not to scale). **Figure S2.** Spheroid growth over several days and with varying starting cell number, shown from day 3 after spheroid preparation. Transmitted light confocal images, 5X objective (each image is 750x750 um). **Figure S3.** Seahorse XFp is a viable option for low replicate studies of spheroid metabolism, depending on spheroid morphology. OCR data from Mito Stress Test of spheroids analyzed in Seahorse XFp are shown compared to data from Seahorse XF96. The OCR values relative to basal do not differ greatly in the CRC models, (A) HCT116 (*n*=4) and (B) SW948 (*n*=5), however PDAC models were more challenging. (C) Signal from Panc1 (*n*=4) may be increased by using larger spheroids or longer culture of the same starting cell number. (D) MIA-Pa-Ca-2 (*n*=3) exhibit a flatter spheroid morphology which presents an issue in the normal Seahorse culture plates, as they lack the special machining of the XF96 spheroid plates that help capture the spheroids and flow fluid around and up the sides of the wells. The movement of these spheroids may be avoided with different coating prior to transfer of the spheroids. **Figure S4.** Protein-normalized OCR and ECAR values in 2D and 3D. (A) OCR from MST in 2D. (B) OCR from MST in 3D. (C) ECAR from GST in 2D. (D) ECAR from GST in 3D. **Figure S5.** Overlaid histograms of protein expression. By cell line, all runs, to show relative magnitudes and expression distribution of the cell populations. Showing normalized peak values to 1000 events. **Figure S6.** Metabolite levels relative to starting amount. (A) Glucose levels in CRC cell lines. (B) Lactate levels in CRC cell lines. (C) Glutamine levels in CRC cell lines. (D) Glucose levels in PDAC cell lines. (B) Lactate levels in PDAC cell lines. (C) Glutamine levels in PDAC cell lines. Blue is 2D, Red is 3D. Error bars represent standard deviation. **Table S1.** Forward scatter intensity from flow cytometry experiments. Cells from spheroids exhibit lower intensity of forward scattering and a higher coefficient of variation. Experimental replicates 1 and 2 were performed on BioRad S3e and replicate 3 on CytoFlex. The large difference in forward scattering magnitudes between these runs are due to the difference in detector sensitivities between the instruments. **Table S2.** Seahorse CCCP concentrations. Final concentration of CCCP in the wells during Seahorse assays for each cell line in 2D and 3D, based on titration over a range of concentrations yielding the maximum OCR values.

## Data Availability

The datasets generated and/or analyzed during the current study are available in the Figshare repository, 10.6084/m9.figshare.15121272.v2.

## Abbreviations

2D: Two-dimensional
3D: Three-dimensional
ATP: Adenosine triphosphate
CCCP: Carbonyl cyanide m-chlorophenyl hydrazone
CRC: Colorectal cancer
ECAR: Extracellular acidification rate
GST: Glycolysis stress test
MST: Mitochondrial stress test
OCR: Oxygen consumption rate
OXPHOS: Oxidative phosphorylation
PDAC: Pancreatic ductal adenocarcinoma
PPR: Proton production rate

## References

[1] Warburg O (1925). The metabolism of carcinoma cells. J Cancer Res.

[2] Hanahan D, Weinberg RA (2011). Hallmarks of cancer: the next generation. Cell.

[3] Luengo A, Gui DY, Vander Heiden MG (2017). Targeting metabolism for cancer therapy. Cell Chem Biol.

[4] DeBerardinis RJ, Chandel NS (2016). Fundamentals of cancer metabolism. Sci Adv.

[5] Faubert B, DeBerardinis RJ (2017). Analyzing tumor metabolism in vivo. Annu Rev Cancer Biol.

[6] Hensley CT (2016). Metabolic heterogeneity in human lung tumors. Cell.

[7] Muir A, Vander Heiden MG (2018). The nutrient environment affects therapy. Science (New York, N.Y.).

[8] Niepel M (2019). A Multi-center study on the reproducibility of drug-response assays in mammalian cell lines. Cell Syst.

[9] Grimes DR (2016). The role of oxygen in avascular tumor growth. PloS One.

[10] Folkman J, Folkman J (2000). Tumor angiogenesis. Holland-Frei Cancer Medicine.

[11] Torrence ME, Manning BD (2018). Nutrient sensing in cancer. Annu Rev Cancer Biol.

[12] Leary E, Rhee C, Wilks B, Morgan JR (2016). Accurate quantitative wide-field fluorescence microscopy of 3-D spheroids. BioTechniques.

[13] Sutherland RM, McCredie JA, Inch WR (1971). Growth of multicell spheroids in tissue culture as a model of nodular carcinomas. J Natl Cancer Inst.

[14] Ayuso JM (2018). Organotypic microfluidic breast cancer model reveals starvation-induced spatial-temporal metabolic adaptations. EBioMedicine.

[15] Schroll MM, LaBonia GJ, Ludwig KR, Hummon AB (2017). Glucose restriction combined with autophagy inhibition and chemotherapy in HCT 116 spheroids decreases cell clonogenicity and viability regulated by tumor suppressor genes. J Proteome Res.

[16] Fan TW-M, et al. Stable isotope-resolved metabolomics shows metabolic resistance to anti-cancer selenite in 3D spheroids versus 2D cell cultures. Metabolites. 2018;8. 10.3390/metabo8030040. 10.3390/metabo8030040 PMC6161115 29996515

[17] Noel P, et al. Preparation and metabolic assay of 3-dimensional spheroid co-cultures of pancreatic cancer cells and fibroblasts. J Vis Exp. 2017. 10.3791/56081. 10.3791/56081 PMC5614363 28872142

[18] Russell S, Wojtkowiak J, Neilson A, Gillies RJ. Metabolic profiling of healthy and cancerous tissues in 2D and 3D. Sci Rep. 2017;7(15285). 10.1038/s41598-017-15325-5. 10.1038/s41598-017-15325-5 PMC5681543 29127321

[19] Alsahli M, Gerich JE, Huhtaniemi I, Martini L (2018). Normal glucose physiology. Encyclopedia of Endocrine Diseases.

[20] Daemen A (2015). Metabolite profiling stratifies pancreatic ductal adenocarcinomas into subtypes with distinct sensitivities to metabolic inhibitors. Proc Natl Acad Sci U S A.

[21] Romero N, Swain PM, Kam Y, Rogers G, Dranka BP. Poster #3487: Bioenergetic profiling of cancer cell lines: quantifying the impact of glycolysis on cell proliferation. AACR Annual Meeting. 2018.

[22] Han C, Takayama S, Park J. Formation and manipulation of cell spheroids using a density adjusted PEG/DEX aqueous two phase system. Sci Rep. 2015;5(11891). 10.1038/srep11891. 10.1038/srep11891 PMC4491721 26144552

[23] Hoffmann OI (2015). Impact of the spheroid model complexity on drug response. J Biotechnol.

[24] Lao Z (2015). Improved methods to generate spheroid cultures from tumor cells, tumor cells & fibroblasts or tumor-fragments: microenvironment, microvesicles and MiRNA. PloS One.

[25] Lee DW (2018). Pitch-tunable pillar arrays for high-throughput culture and immunohistological analysis of tumor spheroids. RSC Adv.

[26] Wen Z (2013). A spheroid-based 3-D culture model for pancreatic cancer drug testing, using the acid phosphatase assay. Braz J Med Biol Res.

[27] Feist PE, Sun L, Liu X, Dovichi NJ, Hummon AB (2015). Bottom-up proteomic analysis of single HCT 116 colon carcinoma multicellular spheroids. Rapid Commun Mass Spectrom.

[28] Paškevičiūtė M, Petrikaitė V (2017). Differences of statin activity in 2D and 3D pancreatic cancer cell cultures. Drug Des Dev Ther.

[29] Gaviraghi M (2011). Pancreatic cancer spheres are more than just aggregates of stem marker-positive cells. Biosci Rep.

[30] Mookerjee SA, Gerencser AA, Nicholls DG, Brand MD (2017). Quantifying intracellular rates of glycolytic and oxidative ATP production and consumption using extracellular flux measurements. J Biol Chem.

[31] Mookerjee SA, Goncalves RLS, Gerencser AA, Nicholls DG, Brand MD (2015). The contributions of respiration and glycolysis to extracellular acid production. Biochim Biophys Acta.

[32] Lue H-W (2017). Metabolic reprogramming ensures cancer cell survival despite oncogenic signaling blockade. Genes Dev.

[33] Damiani C (2017). A metabolic core model elucidates how enhanced utilization of glucose and glutamine, with enhanced glutamine-dependent lactate production, promotes cancer cell growth: The WarburQ effect. PLoS Comput Biol.

[34] Vyas M (2019). Glucose metabolic reprogramming and cell proliferation arrest in colorectal micropapillary carcinoma. Gastroenterol Res.

[35] Rousset M, Dussaulx E, Chevalier G, Zweibaum A (1980). Growth-related glycogen levels of human intestine carcinoma cell lines grown in vitro and in vivo in nude mice. J Natl Cancer Inst.

[36] Alhourani AH, et al. Metformin treatment response is dependent on glucose growth conditions and metabolic phenotype in colorectal cancer cells. Sci Rep. 2021;11. 10.1038/s41598-021-89861-6. 10.1038/s41598-021-89861-6 PMC8131751 34006970

[37] Masoud R (2020). Targeting mitochondrial complex I overcomes chemoresistance in high OXPHOS pancreatic cancer. Cell Rep Med.

[38] Wrzesinski K (2014). The cultural divide: exponential growth in classical 2D and metabolic equilibrium in 3D environments. PloS One.

[39] Kasinskas RW, Venkatasubramanian R, Forbes NS (2014). Rapid uptake of glucose and lactate, and not hypoxia, induces apoptosis in three-dimensional tumor tissue culture. Integrative Biol.

[40] Jagiella N, Müller B, Müller M, Vignon-Clementel IE, Drasdo D (2016). Inferring growth control mechanisms in growing multi-cellular spheroids of NSCLC cells from spatial-temporal image data. PloS Comput Biol.

[41] Jiang L, et al. Reductive carboxylation supports redox homeostasis during anchorage-independent growth. Nature. 2016;532(255–258). 10.1038/nature17393. 10.1038/nature17393 PMC4860952 27049945

[42] Dranka, B. P. et al. Abstract B07: Metabolic liabilities of human colon carcinoma spheroids are different compared to standard 2D cultures. In Tumor Microenvironment and Metabolic Adaptation (American Association for Cancer Research01012015), B07-B07.

[43] Longati P (2013). 3D pancreatic carcinoma spheroids induce a matrix-rich, chemoresistant phenotype offering a better model for drug testing. BMC Cancer.

[44] Muciño-Olmos EA (2020). Unveiling functional heterogeneity in breast cancer multicellular tumor spheroids through single-cell RNA-seq. Sci Rep.

[45] Park S-H (2019). TOMM20 as a potential therapeutic target of colorectal cancer. BMB Rep.

[46] Baek G (2014). MCT4 defines a glycolytic subtype of pancreatic cancer with poor prognosis and unique metabolic dependencies. Cell Rep.

[47] Curry JM (2013). Cancer metabolism, stemness and tumor recurrence: MCT1 and MCT4 are functional biomarkers of metabolic symbiosis in head and neck cancer. Cell cycle (Georgetown, Tex.).

[48] Whitaker-Menezes D (2011). Evidence for a stromal-epithelial "lactate shuttle" in human tumors: MCT4 is a marker of oxidative stress in cancer-associated fibroblasts. Cell cycle (Georgetown, Tex.).

[49] San-Millán I, Julian CG, Matarazzo C, Martinez J, Brooks GA (2019). Is lactate an oncometabolite? Evidence supporting a role for lactate in the regulation of transcriptional activity of cancer-related genes in MCF7 breast cancer cells. Front Oncol.

[50] Macheda ML, Rogers S, Best JD (2005). Molecular and cellular regulation of glucose transporter (GLUT) proteins in cancer. J Cell Physiol.

[51] Szablewski L (2013). Expression of glucose transporters in cancers. Biochim Biophys Acta.

[52] Dyrstad SE, et al. Blocking aerobic glycolysis by targeting pyruvate dehydrogenase kinase in combination with EGFR TKI and ionizing radiation increases therapeutic effect in non-small cell lung cancer cells. Cancers. 2021;13. 10.3390/cancers13050941. 10.3390/cancers13050941 PMC7956357 33668151

[53] Medina RA, Owen GI (2002). Glucose transporters: expression, regulation and cancer. Biol Res.

[54] Gould GW, Holman GD (1993). The glucose transporter family: structure, function and tissue-specific expression. Biochem J.

[55] Gorovits N, Charron MJ (2003). What we know about facilitative glucose transporters: Lessons from cultured cells, animal models, and human studies. Biochem Mol Biol Educ.

[56] Gao H (2020). Prognostic value of glucose transporter 3 expression in hepatocellular carcinoma. Oncol Lett.

[57] Mueller-Klieser W, Freyer JP, Sutherland RM (1986). Influence of glucose and oxygen supply conditions on the oxygenation of multicellular spheroids. Br J Cancer.

[58] Gatenby RA (2007). Cellular adaptations to hypoxia and acidosis during somatic evolution of breast cancer. Br J Cancer.

[59] Pujol-Gimenez J (2015). Could GLUT12 be a potential therapeutic target in cancer treatment? A preliminary report. J Cancer.

[60] Freyer JP, Sutherland RM (1980). Selective dissociation and characterization of cells from different regions of multicell tumor spheroids. Cancer Res.

[61] Rousset S (2007). UCP2 is a mitochondrial transporter with an unusual very short half-life. FEBS Lett.

[62] Cantor JR (2017). Physiologic medium rewires cellular metabolism and reveals uric acid as an endogenous inhibitor of UMP synthase. Cell.

[63] Vande Voorde J (2019). Improving the metabolic fidelity of cancer models with a physiological cell culture medium. Sci Advanc.

