# Supplementary material for: Metabolic flux analysis of 3D spheroids reveals significant differences in glucose metabolism from matched 2D cultures of colorectal cancer and pancreatic ductal adenocarcinoma cell lines
Source: Cancer Metab. 2022 May 16;10:9. doi: 10.1186/s40170-022-00285-w (PMC9109327; doi:10.1186/s40170-022-00285-w)
Supplement: Supplementary file 1 — Additional file 1: Figure S1. Morphology of cell lines. 2D adherent (top) and 3D spheroid (bottom) cultures. (Spheroid size for detail, not to scale). Figure S2. Spheroid growth over several days and with varying starting cell number, shown from day 3 after spheroid preparation. Transmitted light confocal images, 5X objective (each image is 750x750 um). Figure S3. Seahorse XFp is a viable option for low replicate studies of spheroid metabolism, depending on spheroid morphology. OCR data from Mito Stress Test of spheroids analyzed in Seahorse XFp are shown compared to data from Seahorse XF96. The OCR values relative to basal do not differ greatly in the CRC models, (A) HCT116 (n=4) and (B) SW948 (n=5), however PDAC models were more challenging. (C) Signal from Panc1 (n=4) may be increased by using larger spheroids or longer culture of the same starting cell number. (D) MIA-Pa-Ca-2 (n=3) exhibit a flatter spheroid morphology which presents an issue in the normal Seahorse culture plates, as they lack the special machining of the XF96 spheroid plates that help capture the spheroids and flow fluid around and up the sides of the wells. The movement of these spheroids may be avoided with different coating prior to transfer of the spheroids. Figure S4. Protein-normalized OCR and ECAR values in 2D and 3D. (A) OCR from MST in 2D. (B) OCR from MST in 3D. (C) ECAR from GST in 2D. (D) ECAR from GST in 3D. Figure S5. Overlaid histograms of protein expression. By cell line, all runs, to show relative magnitudes and expression distribution of the cell populations. Showing normalized peak values to 1000 events. Figure S6. Metabolite levels relative to starting amount. (A) Glucose levels in CRC cell lines. (B) Lactate levels in CRC cell lines. (C) Glutamine levels in CRC cell lines. (D) Glucose levels in PDAC cell lines. (B) Lactate levels in PDAC cell lines. (C) Glutamine levels in PDAC cell lines. Blue is 2D, Red is 3D. Error bars represent standard deviation. Table S1. Forward sca [file 40170_2022_285_MOESM1_ESM.docx]

## Supplementary Data


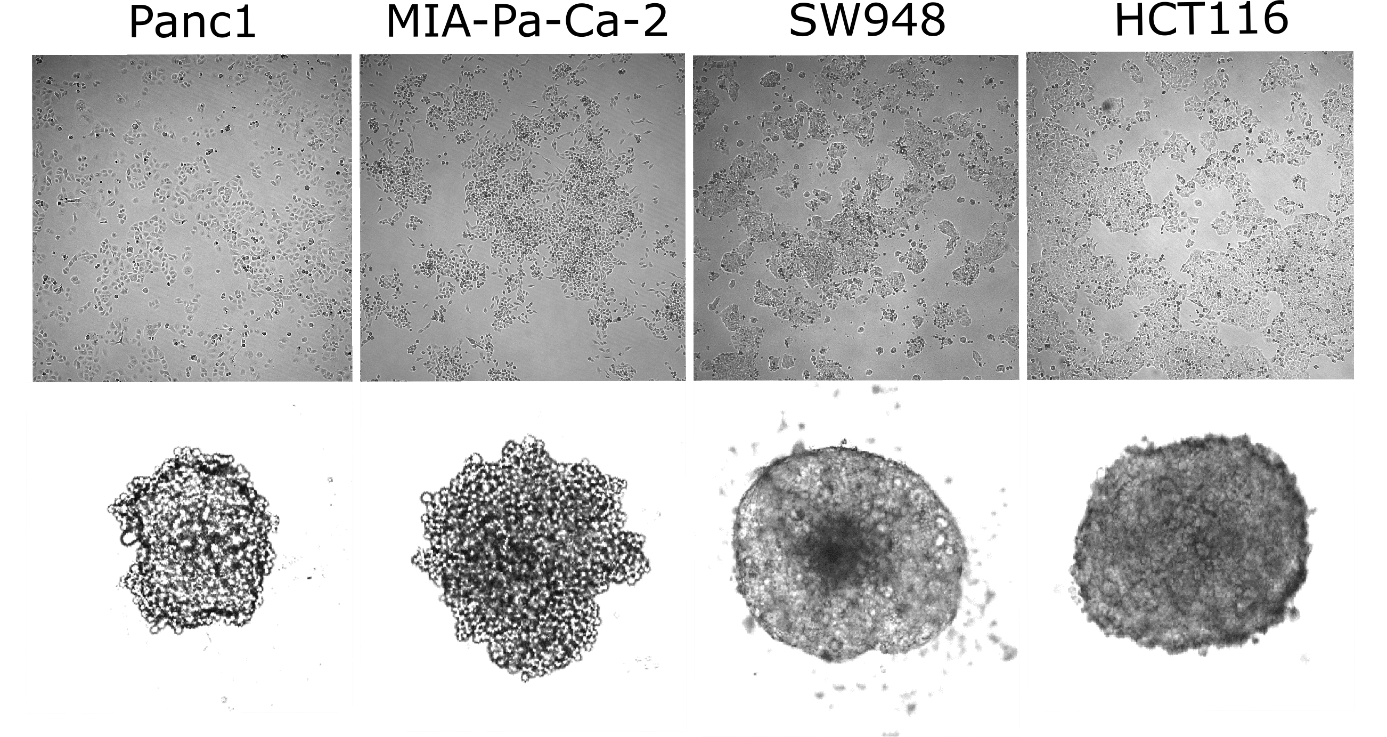


**Figure S1: Morphology of cell lines**. **2D adherent (top) and 3D spheroid (bottom) cultures. (Spheroid size for detail, not to scale)**


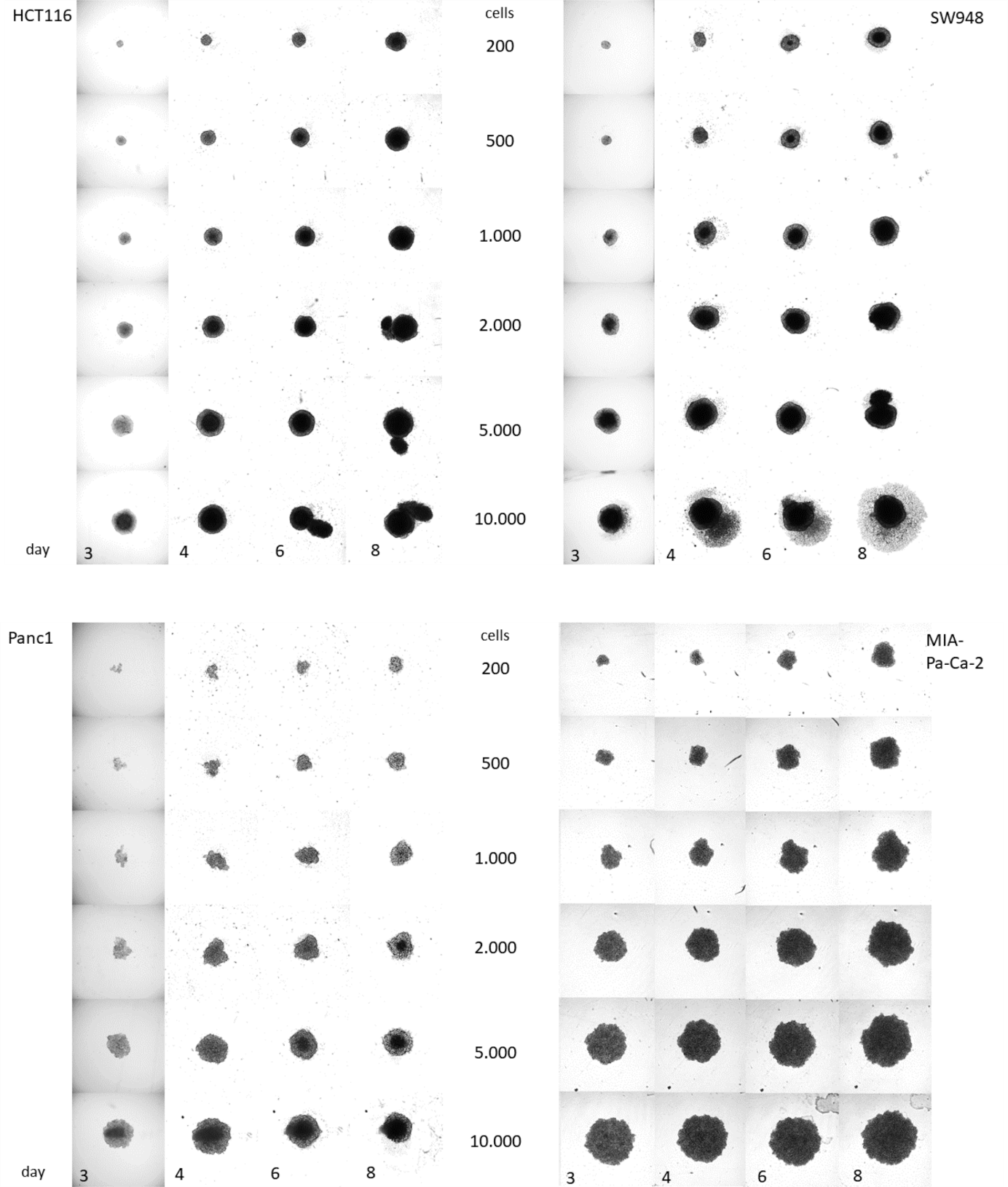


**Figure S2: Spheroid growth over several days and with varying starting cell number, shown from day 3 after spheroid preparation.** **Transmitted light confocal images, 5X objective (each image is 750x750 um).**


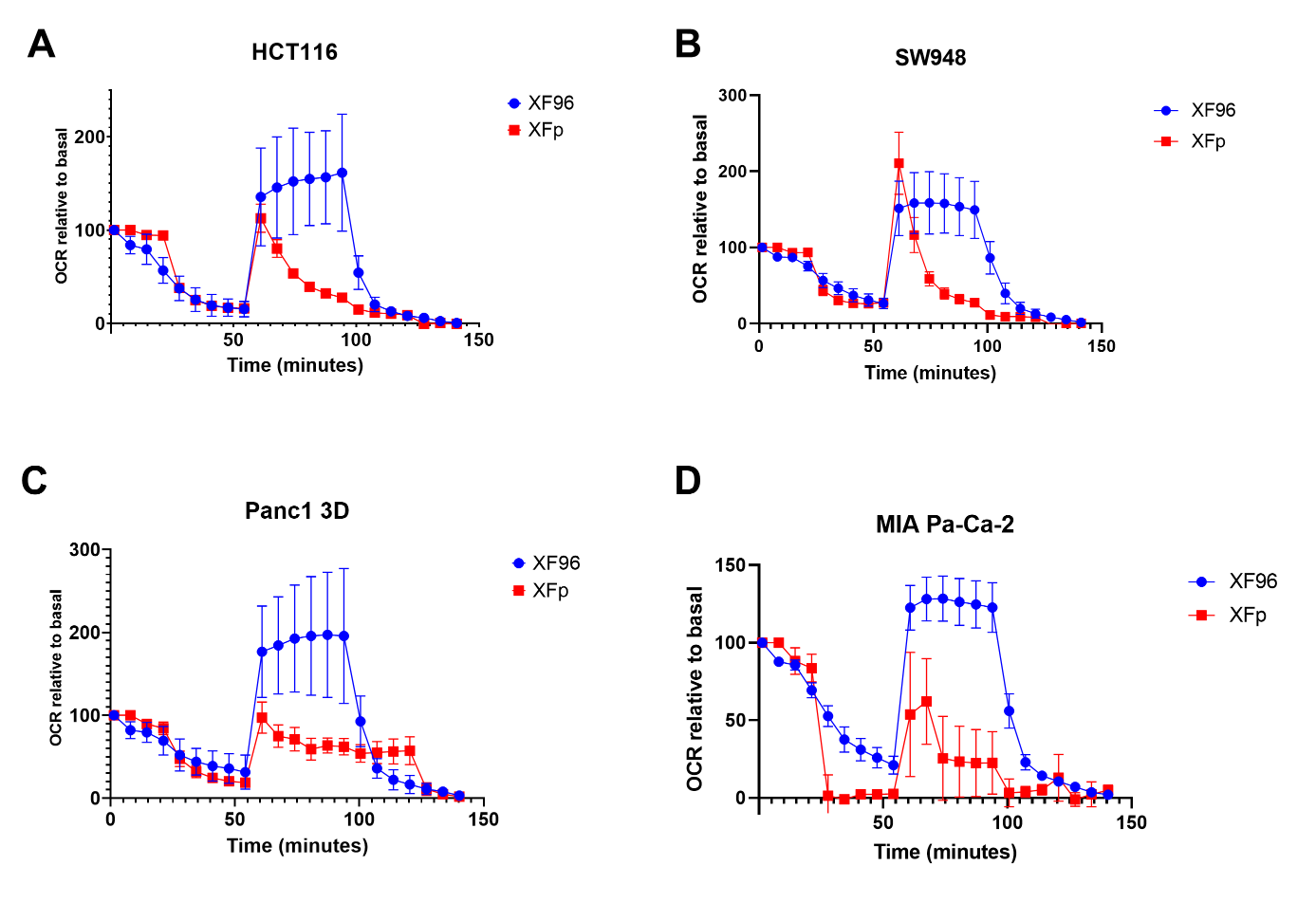


**Figure S3. Seahorse XFp is a viable option for low replicate studies of spheroid metabolism, depending on spheroid morphology.** **OCR data from Mito Stress Test of spheroids analyzed in Seahorse XFp are shown compared to data from Seahorse XF96. The OCR values relative to basal do not differ greatly in the CRC models, (A) HCT116 (n=4) and (B) SW948 (n=5), however PDAC models were more challenging. (C) Signal from Panc1 (n=4) may be increased by using larger spheroids or longer culture of the same starting cell number. (D) MIA-Pa-Ca-2 (n=3) exhibit a flatter spheroid morphology which presents an issue in the normal Seahorse culture plates, as they lack the special machining of the XF96 spheroid plates that help capture the spheroids and flow fluid around and up the sides of the wells. The movement of these spheroids may be avoided with different coating prior to transfer of the spheroids.**


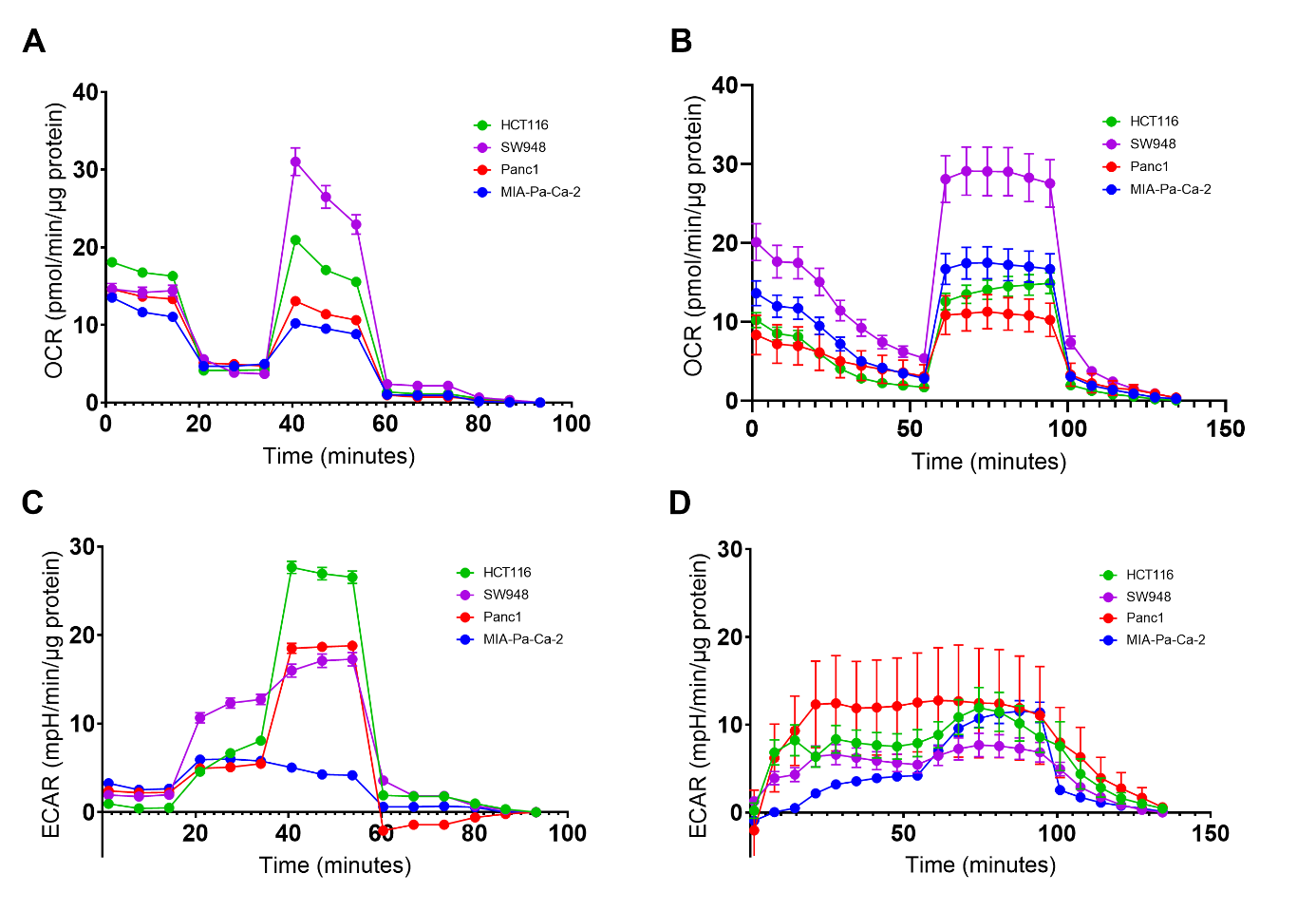


**Figure S4.** **Protein-normalized OCR and ECAR values in 2D and 3D.** **(A) OCR from MST in 2D. (B) OCR from MST in 3D. (C) ECAR from GST in 2D. (D) ECAR from GST in 3D.**


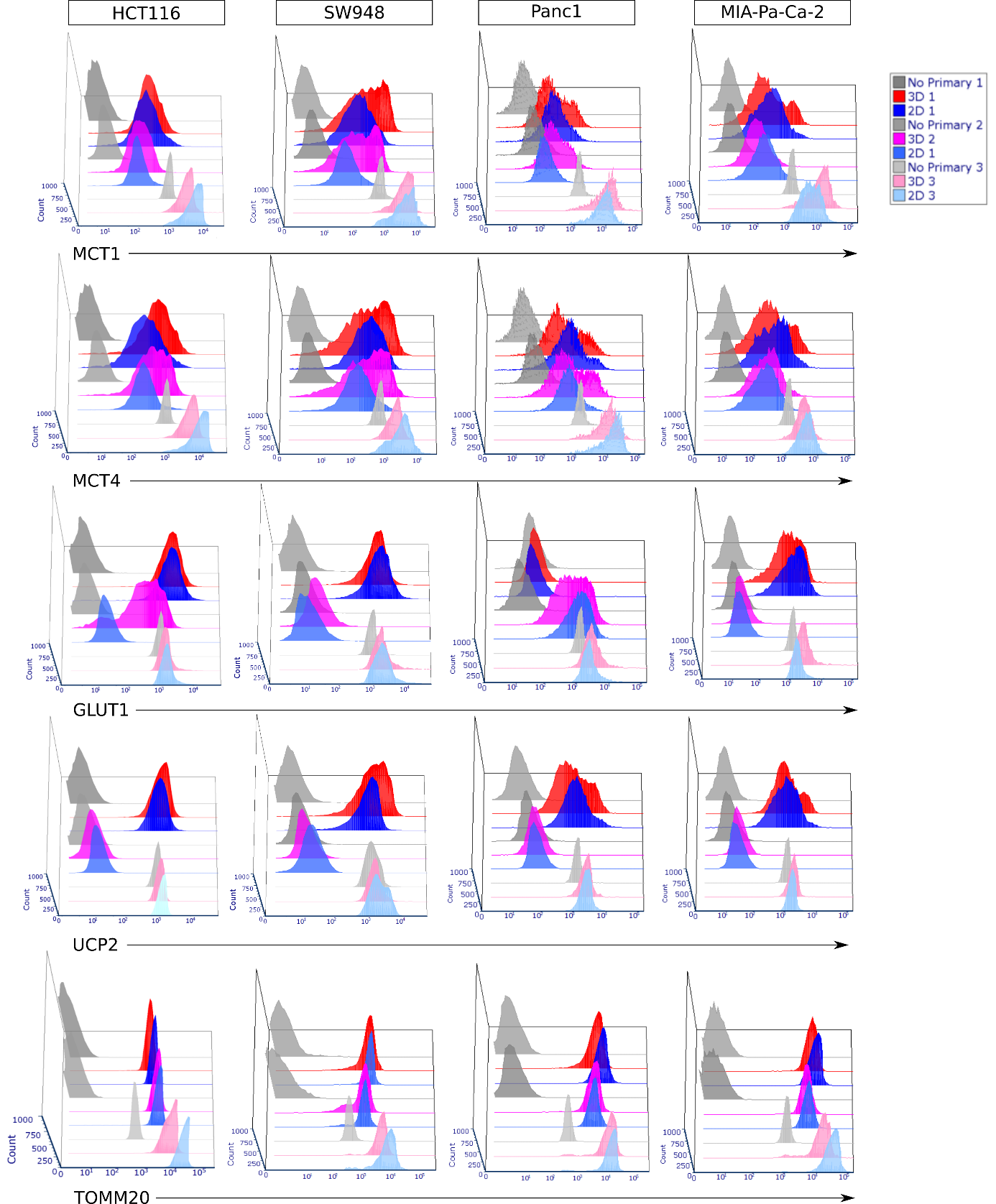


**Figure S5. Overlaid histograms of protein expression**. **By cell line, all runs, to show relative magnitudes and expression distribution of the cell populations. Showing normalized peak values to 1000 events.**


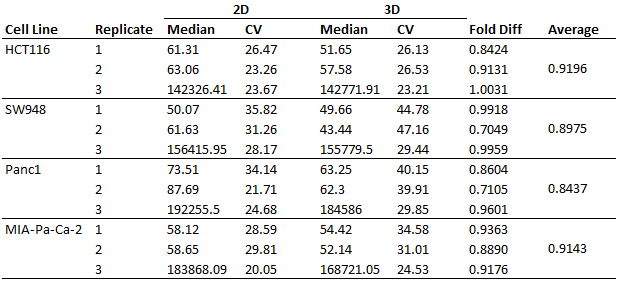


**Table S1. Forward scatter intensity from flow cytometry experiments**. **Cells from spheroids exhibit lower intensity of forward scattering and a higher coefficient of variation. Experimental replicates 1 and 2 were performed on BioRad S3e and replicate 3 on CytoFlex. The large difference in forward scattering magnitudes between these runs are due to the difference in detector sensitivities between the instruments.**

| **Cell line** | **2D CCCP** | **3D CCCP** |
| --- | --- | --- |
| HCT116 | 0.5 uM | 4 uM |
| SW948 | 0.5 uM | 3 uM |
| PANC1 | 0.5 uM | 4 uM |
| MIA PACA-2 | 0.5 uM | 2 uM |

**Table S2. Seahorse CCCP concentrations. Final concentration of CCCP in the wells during Seahorse assays for each cell line in 2D and 3D, based on titration over a range of concentrations yielding the maximum OCR values.**


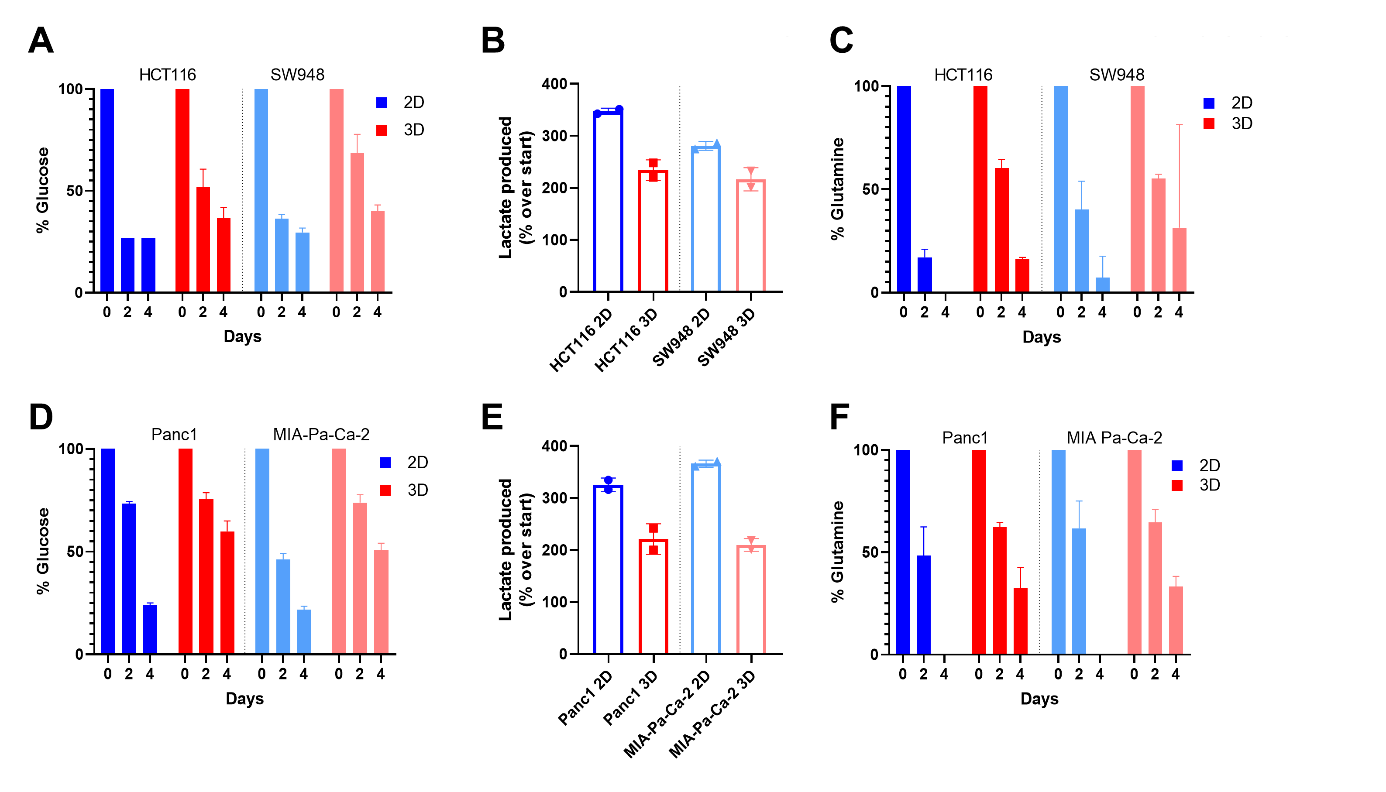


**Figure S6. Metabolite levels relative to starting amount. (A) Glucose levels in CRC cell lines. (B) Lactate levels in CRC cell lines. (C) Glutamine levels in CRC cell lines. (D) Glucose levels in PDAC cell lines. (B) Lactate levels in PDAC cell lines. (C) Glutamine levels in PDAC cell lines. Blue is 2D, Red is 3D. Error bars represent standard deviation.**
